# The effects of semaphorin 3A in bone and cartilage metabolism: fundamental mechanism and clinical potential

**DOI:** 10.3389/fcell.2023.1321151

**Published:** 2023-11-23

**Authors:** KaiLe Wu, Donghua Huang, Xin Huang

**Affiliations:** ^1^ School of Medicine, Second Affiliated Hospital, Zhejiang University, Hangzhou, China; ^2^ Orthopedics Research Institute of Zhejiang University, Hangzhou, China; ^3^ Key Laboratory of Motor System Disease Research and Precision Therapy of Zhejiang Province, Hangzhou, China; ^4^ Clinical Research Center of Motor System Disease of Zhejiang Province, Hangzhou, China

**Keywords:** semaphorin 3A, bone, cartilage, metabolism, tissue engineering

## Abstract

Semaphorin 3A (Sema3A) is a neuroinformatic protein molecule with widespread expression across various tissues and organs. Recent investigations have unveiled its pivotal role in the skeletal system, primarily through its binding interactions with two co-receptors, neuropilin-1 (Nrp-1) and members of the plexin family. Prior research has confirmed the expression of Sema3A and its receptors in both osteocytes and chondrocytes. Beyond its expression patterns, Sema3A plays a multifaceted role in regulating bone and cartilage metabolism via employing diverse signaling pathways. Additionally, it engages in collaborative interactions with the immune and nervous systems, contributing to the pathophysiological processes underlying a spectrum of bone and joint diseases. In this paper, we undertake a comprehensive review of recent research developments in this field. Our objective is to deepen the understanding of Sema3A within the context of skeletal physiology and pathology. Furthermore, we aim to furnish a valuable reference for potential therapeutic interventions in the realm of bone and joint diseases.

## 1 Introduction

### 1.1 Semaphorin and its superfamily

Semaphorins constitute a diverse class of glycoproteins, existing in both secretory and transmembrane forms, and represent the largest family of guidance cues orchestrating the navigation of growth cones within axonal terminals (Fiore and Puschel, 2003). Based on the meticulous analysis of structural domains and the identification of class-specific carboxyl-terminal domains (CTDs), the expansive semaphorin family, comprising over 30 members, has been systematically classified into eight distinct groups (Mark et al., 1997; Goodman et al., 1999). Of particular interest within this array of semaphorins is the class 3 semaphorin, also recognized as collapsin-1 (Sema III/coll-1), notable for its unique structural domain located at the extreme carboxyl terminus (Messersmith et al., 1995). This distinctive structural feature assumes paramount significance as it exerts a profound regulatory influence on a spectrum of fundamental physiological processes. Specifically, it plays a pivotal role in governing angiogenesis (Vacca et al., 2006), modulating immune responses (Takamatsu and Kumanogoh, 2012), directing axon guidance (Polleux et al., 2000), and influencing tumor (Trusolino and Comoglio, 2002). Thus, class 3 semaphorins occupy a central and commanding role within the intricate web of molecular regulators governing these critical biological phenomena.

### 1.2 Sema3A and its receptor

**FIGURE 1:**
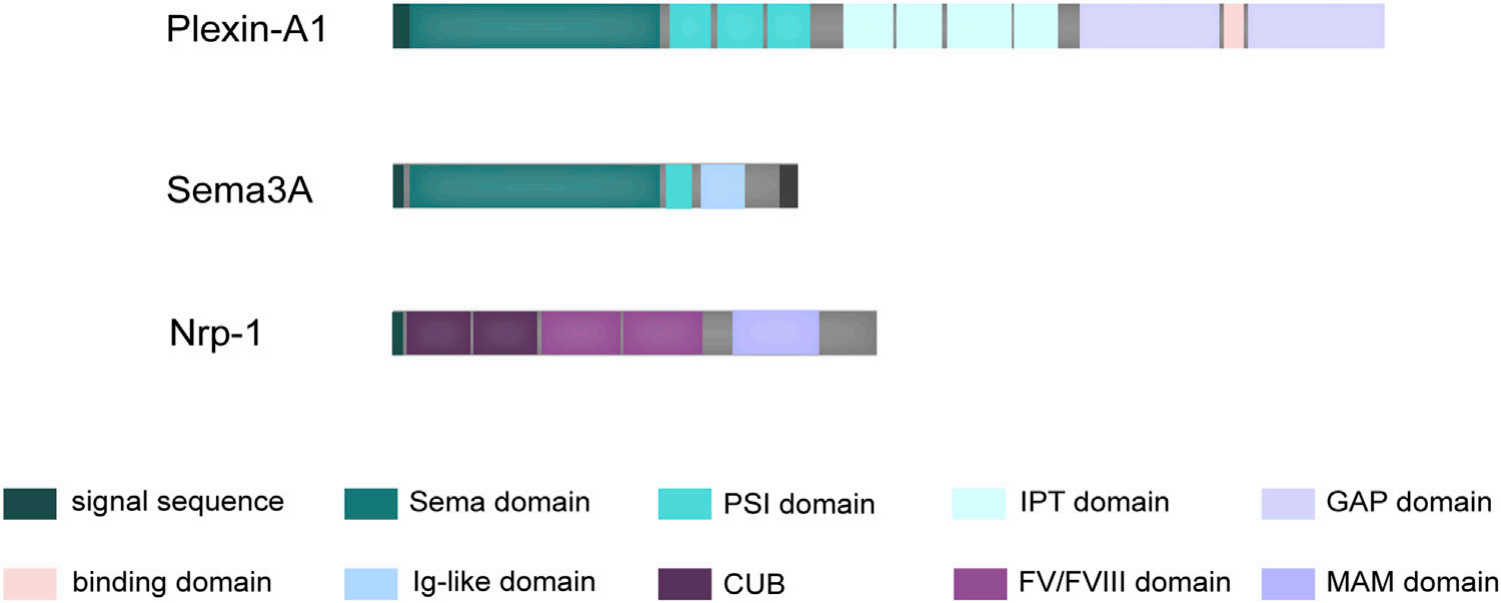
Schematic structure of Sema3A and its receptors. Sema3A is defined by a distinctive structural composition, commencing with the Sema domain, subsequently followed by the PSI domain, and augmented by an additional Ig-like domain. Plexin-A1 possesses a structural arrangement beginning with an N-terminal Sema domain, followed by PSI domains, along with IPT domains in its extracellular region. Within its intracellular domain, Plexin-A1 encompasses two discrete cytoplasmic GTPase-activating protein (GAP) domains. In contrast, Nrp-1 consists of a generous extracellular domain, a solitary transmembrane domain, and a brief cytoplasmic tail. The extracellular segment is characterized by two initial CUB domains, followed by two coagulation FV/FVIII homology domains, and concludes with a membrane-proximal MAM domain.

Diffusible axon chemotaxis is facilitated by Semaphorin 3A (Sema3A), the first member of a sizable family of signaling peptides to be found. Beyond its well-documented involvement in various physiopathological processes, it has drawn attention to its protective function in bone homeostasis (Polleux et al., 2000; Hayashi et al., 2012). A distinguishing structural feature that defines the function of semaphorin superfamily proteins is the presence of the sema domain, followed by a short plexin–semaphorin–integrin (PSI) domain (Figure 1). This sema domain, characterized by a slightly modified version of the largely conserved seven-blade β-propeller fold, assumes a critical role in mediating the dimerization and receptor-ligand binding of these proteins (Gherardi et al., 2004). Complementing its fundamental structure, Sema3A is also endowed with an Ig-like domain that exhibits potential involvement in immune responses (Love et al., 2003).

Physiologically, Sema3A exerts its influence through binding to two co-transmembrane receptors, neuropilin-1 (Nrp-1) and Plexin-A family, resulting in the formation of a functional Sema3A/Nrp-1/Plexin-A complex (Rohm et al., 2000). The Plexin-A family encompasses Plexin-A1, Plexin-A2, Plexin-A3, and Plexin-A4, characterized by a highly conserved SP (Sex and Plexins) cytoplasmic domain consisting of 600 amino acids, which potentially serves as a site for tyrosine phosphorylation. Differential Plexin-A binding leads to the formation of complexes with varying physiological effects. Current research on the receptors involved in the osteoprotective mechanism of Sema3A has primarily focused on Plexin-A1. However, it is important to note that the Plexin-A family is interconnected, and silencing some receptors can be compensated for by the overexpression of others. For instance, overexpressing Plexin-A2 in Plexin-A1 or Plexin-A4 silenced endothelial cells can restore their response to Sema3A (Sabag et al., 2014). Additionally, the *PLXNA2*, which encodes Plexin-A2, has been identified in human chondrocytes as a potential candidate gene for mandibular proptosis, which suggests that Sema3A may influence human chondrocytes through its interaction with Plexin-A2 (Kajii et al., 2018).

Notably, the interaction between Sema3A and Plexin-A, while direct, tends to be relatively weak, a characteristic attributed to their structural homology (Antipenko et al., 2003). When evaluated individually, the short cytoplasmic tails of Nrp-1 fail to confer biological activity comparable to that of Plexin-A (Tamagnone et al., 1999). Nrp-1, a cell surface glycoprotein, features extracellular structural domains, including two N-terminal CUB domains (a1 and a2), two coagulation factor V/VIII homologous domains (b1 and b2), and a near-membrane MAM domain (domain c) (Figure 1). Domain a1, in particular, plays a crucial role in cross-supporting the Sema3A-Plexin A1 complex, effectively locking the structure of the co-receptor complex (Janssen et al., 2012). Interestingly, it has also been elucidated that the Nrp-1 possesses a Sema3A binding site, closely resembling the dimerization interface of Sema3A (Antipenko et al., 2003). Consequently, the signaling is mediated through a precise and dynamic ligand-receptor interaction.

### 1.3 Downstream signaling of Sema3A and its receptor

Numerous studies have illuminated the critical role of canonical Wnt signaling in osteogenic processes, underlining its strong association with bone homeostasis and raising intriguing questions about the potential involvement of Sema3A as a pivotal trigger (Baron and Kneissel, 2013). A recent investigation by Xie and colleagues has shed light on the regulatory role of miR-196b-5p, implicated in various malignancies, in osteoblast and osteoclast differentiation. Their findings suggest that miR-196b-5p exerts its influence by targeting Sema3A and impeding the Wnt-β-catenin signaling pathway (Xie et al., 2023). However, the role of the Wnt signaling system in osteogenic differentiation remains a subject of debate. While some studies have emphasized its significance, others have revealed that this pathway can paradoxically inhibit osteogenic development (Cho et al., 2006). This intriguing duality warrants further exploration.

Besides, Sema3A regulates osteoclastic differentiation by impeding the tyrosine phosphorylation of phospholipase Cγ2 (PLCγ2) induced by receptor activator of nuclear factor kappa-B ligand (RANKL). This regulation occurs through the downstream immunoreceptor tyrosine-based activation motif (ITAM) and RhoA/ROCK signaling pathways (Hayashi et al., 2012; Tse, 2012). Key signaling pathways, including nuclear factor of activated T cells, cytoplasmic 1 (NFATc1), nuclear factor kappa B (NF-κB), and Akt/protein kinase B (PKB), are well-established in osteoclasts, activated by the RANKL/RANK axis (Boyle et al., 2003). Remarkably, Sema3A and neuropilin-1 (Nrp-1) contribute to the fine-tuning of NFATc1 activation through a complex crosstalk involving the ITAM adaptor molecule DNAX-activating protein 12 (DAP12) and RANKL/RANK signaling (Feng, 2005).

### 1.4 Derivation of Sema3A in bone and cartilage metabolism

Sema3A is extensively expressed in the hypothalamus, and it plays a function in axon guidance and neuronal cell migration during the development of the central nervous system, meanwhile, its mutant genes are also considered to be the culprits of obesity, because the paraventricular nucleus and the arcuate nucleus, where Sema3A and its receptors are located, are intimately related to the body’s energy uptake and expenditure, and one of the key factors mediating this is the interaction between neuropeptide Y(NPY) and leptin receptor (OB-R). Interestingly, leptin has long been recognized as a key factor in the central control of bone remodeling, coordinating bone resorption and bone formation via the sympathetic nervous system, whereas NPY has also been proposed to play a role in regulating vasculature, osteoblasts, and osteoclasts (Takeda, 2008; van der Klaauw et al., 2019). Sema3A is also expressed in cartilaginous tissue like articular cartilage (Li et al., 2017), after secretion, Sema3a performs paracrine and autocrine activities (Han et al., 2018). Thus, the purpose of this review is to evaluate the effects of Sema3A on bone and cartilage metabolism in various skeletal ailments, as well as its value in bone tissue engineering, to comprehend its potential as a new therapeutic approach in bone diseases.

## 2 Roles of Sema3A in bone metabolism and diseases

### 2.1 Sema3A signaling in bone

#### 2.1.1 Direct role of Sema3A in bone homeostasis via bone cells

Bone is an extremely dense structure in humans that helps to support body’s weight and preserve its shape. A decrease in bone volume has been observed in lumbar vertebrae and long bones when Sema3A is selectively deleted in osteoblast lineage cells (Yamashita et al., 2022). Similarly, targeted mutation of the *SEMA3A* gene in mice led to vertebral fusion and partial rib duplication, emphasizing its role in skeletal development (Behar et al., 1996). In essence, Sema3A expression plays a pivotal role in promoting bone mass formation and skeletal development.

The human skeleton comprises three primary cell types: osteoblasts, osteocytes, and osteoclasts. Osteoblasts derived from bone marrow stromal cells (BMSCs) differentiate into osteocytes and form bone, whereas osteoclasts derived from bone marrow macrophages and resorb bone. The following is a description of the several ways that Sema3A affects the three different cell types.

##### 2.1.1.1 Osteocytes

The journey of mesenchymal stem cells through osteoblast development culminates in the formation of osteocytes, integral residents within the bone matrix. Osteocytes, far from passive entities, actively contribute to the maintenance of bone homeostasis through a myriad of mechanisms. These encompass the activation of osteoclasts, the orchestration of programmed apoptosis and autophagy, and the meticulous control of bone mineral metabolism (Bonewald, 2011). Nitri coxide synthase (NOS) activation ultimately leads to the activation of protein kinase (PKG), which assumes a pivotal role in suppressing osteocyte cell turnover. Therefore, the NO-sGC-cGMP-PKG pathway is recognized to have a significant role in skeletal dynamic homeostasis (Marathe et al., 2012).

Intriguingly, osteocytes also express Sema3A and its receptors, Hayashi et al. (Hayashi et al., 2019) illuminate that adult mice, characterized by Sema3A deficiency in osteocytes, exhibit diminished bone mass and a reduced population of osteoclasts. Furthermore, *in vitro* experiments unveil the potential of sGC activators to abate bone loss in Sema3A-specific osteoblast lineage knockout mice. From a general point of view, Sema3A was able to activate the sGC-cGMP signaling pathway to protect osteocytes from apoptosis, whereas cGMP was not observed in undifferentiated osteocyte production.

In summary, osteocytes emerge as active regulators within the bone matrix, intricately involved in shaping bone homeostasis. The multifaceted orchestration of the NO-sGC-cGMP-PKG pathway and the elucidation of Sema3A’s signaling underscore the nuanced role of osteocytes in skeletal dynamics.

##### 2.1.1.2 Osteoblasts and osteoclasts

Bone remodeling, a dynamic process essential for skeletal maintenance, hinges on the coordinated efforts of osteoblasts and osteoclasts, orchestrating bone formation and resorption. The role of the axon guidance molecule Semaphorin III in this intricate process has gradually come to light over the years. As early as 2000, Togari et al. (Togari et al., 2000) first unveiled the expression of *sema III* mRNA on both osteoblasts and osteoclasts. However, its precise contribution to bone remodeling remained shrouded in mystery. Subsequent investigations by Takegahara et al. (Takegahara et al., 2006) identified Plexin-A1 as one of the receptors for this enigmatic semaphorin, whereas a reduction in osteoclast numbers was shown in *Plexin-A1* ^*−/−*^ mice, but there was no significant effect on osteoblast numbers. Long bone volume and trabecular mass, however, unexpectedly increased. Meanwhile, osteoclast inhibition was attenuated upon knockdown of Nrp-1 in osteocytes (Azab et al., 2020). In light of these findings, it becomes evident that semaIII and its receptors exert profound effects on bone physiology, mediated through both osteoblasts and osteoclasts.

**FIGURE 2:**
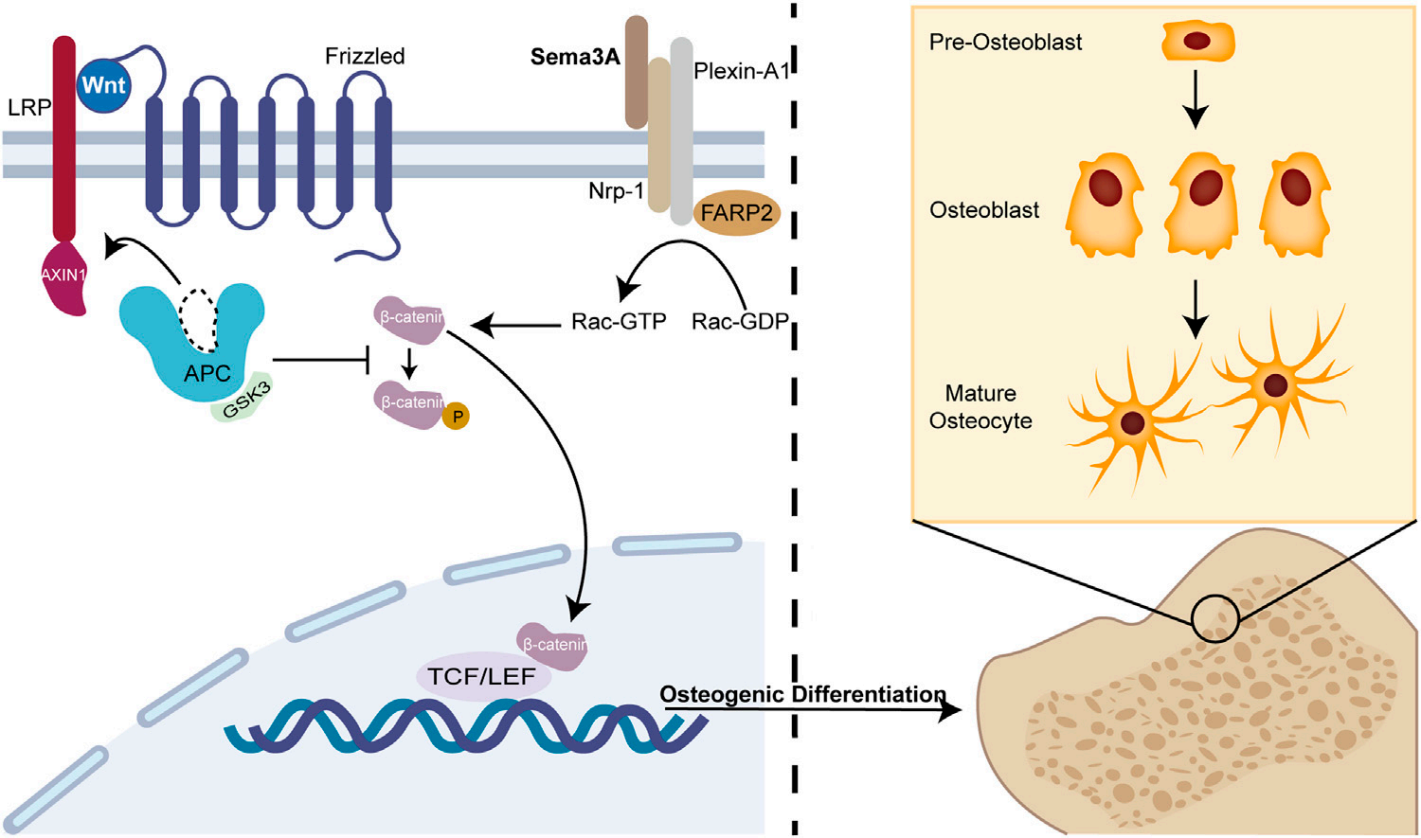
Sema3A promotes osteoblast differentiation. The Wnt/β-catenin signaling system has been linked to osteogenic differentiation in previous studies. In the absence of Wnt, β-catenin undergoes phosphorylation and subsequently becomes sequestered by the destruction complex, a molecular assembly comprising APC, GSK3, and Axin1. Ultimately, it is targeted for degradation by the proteasome. When Sema3A binds to Nrp-1 and Plexin-A1, it activates Rac 1 and the canonical Wnt signaling pathway. This interaction recruits Axin1, leading to the liberation of β-catenin from the cytoplasm when Wnt binds to frizzled. Thus, nuclear β-catenin engages the TCF/LEF complex to enhance osteoblast differentiation, which is an indispensable process in bone remodeling.

The intricate web of molecular players orchestrating osteoblast-osteoclast interactions within the bone microenvironment has long eluded comprehensive understanding. In this context, the revelation of molecules capable of concurrently mediating these interactions through the signaling pathway remained enigmatic. In 2012, Hayashi et al. (Hayashi et al., 2012) unveiled a pivotal role for Sema3A as a local effector that exerts control over bone mass by dual modulation of osteoblasts and osteoclasts. Sema3A acts as a unique regulator, inhibiting osteoclastic bone resorption while concurrently promoting osteoblastic bone formation. In *Sema3A* ^*−/−*^ cells, Wnt signaling pathway-related genes for osteoblasts were markedly downregulated, whereas Wnt3a-induced β-catenin nuclear accumulation was similarly repressed in the canonical Wnt pathway (Figure 2). FARP2, a guanine nucleotide exchange factor (GEF) containing the FERM structural domain, which can interact directly with Plexin-A1 and split apart when Nrp-1 and Sema3A are bound, triggering Rac GEF activation (Toyofuku et al., 2005). Hence, exogenous Sema3A can activate Rac1 to further start the canonical Wnt signaling pathway regulating osteoblast development.

**FIGURE 3:**
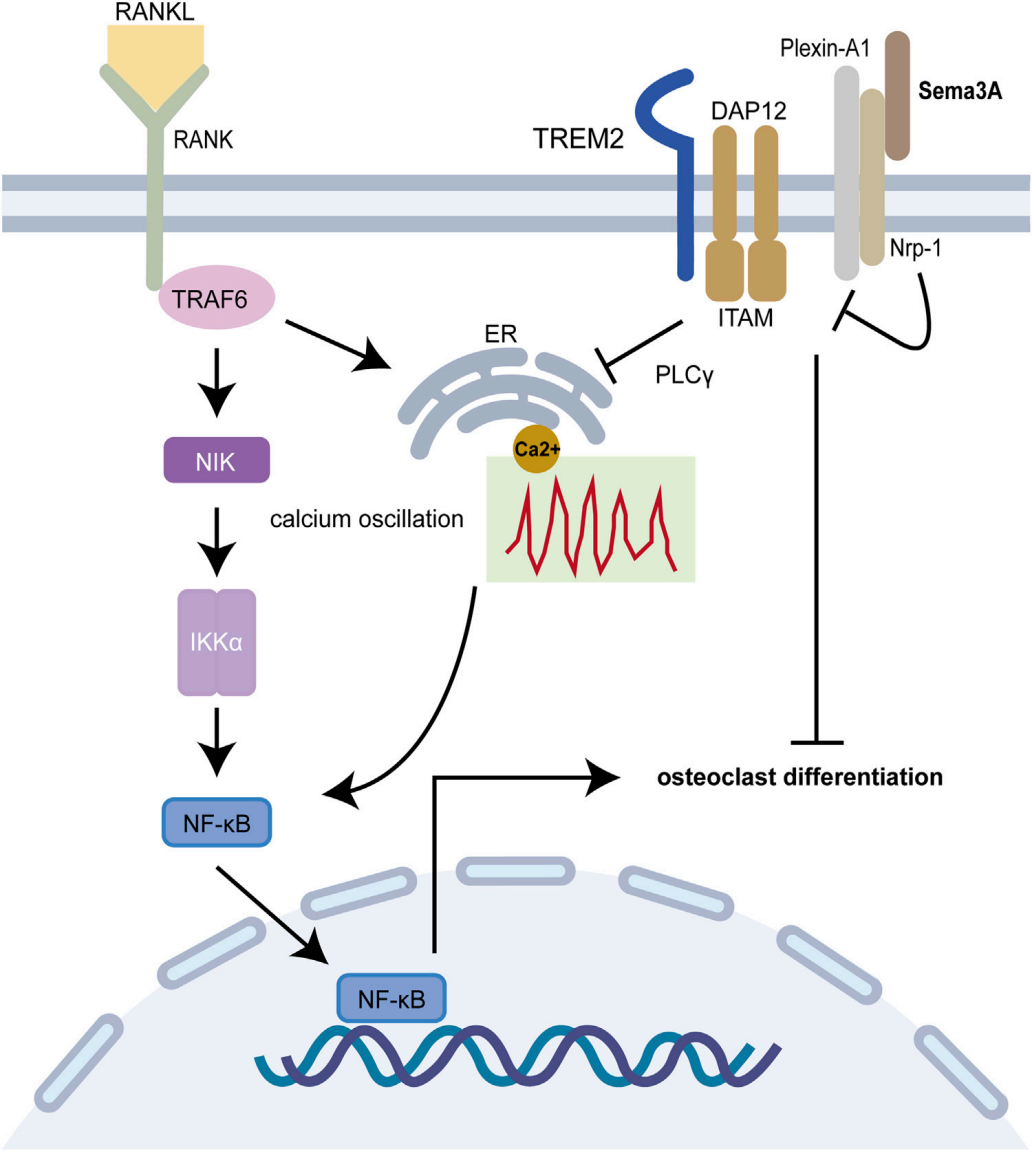
Sema3A inhibits osteoclast differentiation. The RANKL/RANK signaling pathway is required for osteoclast differentiation, and the Plexin A1-TREM2-DAP12 complex can activate ITAM signaling. After enlisting TRAF6, RANK further activates NF-B and takes it into the nucleus to start osteoclast-related genes. Calcium oscillation also plays a crucial role in osteoclast differentiation. In addition to competitively binding to Plexin-A1, Nrp-1 blocks ITAM signaling and calcium oscillations via Sema3A signaling, which in turn prevents osteoclast differentiation and function.

On another front, Plexin A1-TREM2-DAP12 promotes osteoclast differentiation through activating ITAM signaling (Takegahara et al., 2006), but this binding is perturbed by Nrp-1, which releases Plexin-A1 from the complex. However, RANKL/RANK pathway prevents this recruitment, which is reversed by Sema3A treatment (Figure 3). Notably, both ITAM signaling and calcium oscillations, crucial in bone resorption, come into play, with the latter being modulated by an array of agents, including RANKL/RANK, ITAM receptors, and TRP channels (Okada et al., 2021). Interestingly, Fukuda et al. (Fukuda et al., 2013) further proposed that the neuronal source of Sema3A plays a pivotal role in maintaining bone homeostasis. This hints at a dynamic interplay where Sema3A governs bone remodeling and establishes crosstalk with sensory nerves. While neuromodulation of osteogenesis and bone resorption has garnered substantial attention, Fukuda and coworkers underscore the broader scope of Sema3A’s influence (Meyers et al., 2020; Wan et al., 2021). In summary, under physiological circumstances, Sema3A emerges as a regulator, deftly balancing bone production and bone resorption.

The delicate equilibrium between bone formation and resorption plays a pivotal role in maintaining skeletal health. Disruptions in this balance, whether local or systemic, often precipitate pathological consequences. Consequently, researchers have sought to unravel the potential of Sema3A as a therapeutic tool in specific pathological scenarios. Sun et al. (Sun et al., 2021) discovered that Sema3A may still enhance osteoblast differentiation through the canonical Wnt signaling pathway and reduce the suppression of inflammation on the shift of BMSCs towards osteoblast differentiation in a lipopolysaccharide (LPS)-mediated inflammatory environment. In addition, the gradual strain created by the regeneration of local tissues on living tissue results in the existence of stresses, which in turn aid in the active formation of certain tissue structures (Ilizarov, 1989). It is worth noting that Uto and colleagues (Uto et al., 2017) unveiled an intriguing connection between Sema3A and bone mineral density (BMD) around dental implants subjected to mechanical loading. Here, Sema3A emerged as a potential player in enhancing bone quality under stress conditions. The stress condition-specific gene SP7 in alveolar bone osteoblasts upregulates Sema3A, which promotes osteogenic differentiation by binding to Nrp-1 and Plexin-A1, with potential participation of the Rac1GTPase and β-catenin pathways (Sen et al., 2021). However, high levels of β-catenin in the inflammatory milieu reduce osteogenic differentiation of BMSCs by inhibiting the noncanonical Wnt pathway, indicating the complexity of bone homeostatic regulation (Liu et al., 2011).

Osteoblasts and osteoclasts are intricately connected, osteoblasts can produce ligands for RANK and macrophage colony-stimulating factor (M-CSF) (Zhao et al., 2006). RANKL, a transmembrane protein belonging to the tumor necrosis factor (TNF) superfamily, has long been acknowledged for its pivotal role in regulating osteoclast differentiation through the RANK/RANKL signaling pathway, with the concomitant involvement of M-CSF (Kong et al., 1999). These ligands, by binding to specific receptors, initiate downstream signaling pathways, activating transcription factors such as NF-κB, c-Fos (target gene *NFATC1*), and NFATc1, all essential for osteoclast differentiation (Zhao et al., 2006; Asagiri and Takayanagi, 2007). However, osteoblasts do more than only provide the corresponding ligands, Ikebuchi et al. (Ikebuchi et al., 2018) discovered that secreted RANKL from osteoblasts can activate Runt-related transcription factor 2 (Runx2) (Gaur et al., 2005) via reverse signaling, thereby promoting osteoblastic bone formation and serving as an critical element in recognizing osteoblast-osteoclast-coupled signals. Proline-rich motifs in the RANKL tail play a key role in this process. Similarly, Zhao et al. (Zhao et al., 2006) revealed that the NFATc1 target *EFNB2* gene is expressed by osteoclasts and functions in both blocking the c-Fos-NFATc1 osteoclast differentiation chain through reverse signaling and promoteing osteogenesis via the expression of the ephrin receptor family member EphB4 by osteoblasts (Takyar et al., 2013). Therefore, osteoblasts maintain the delicate balance of calcium and phosphorus metabolism and bone homeostasis, ensuring dynamic equilibrium in bone remodeling (Kim and Koh, 2019).

**FIGURE 4:**
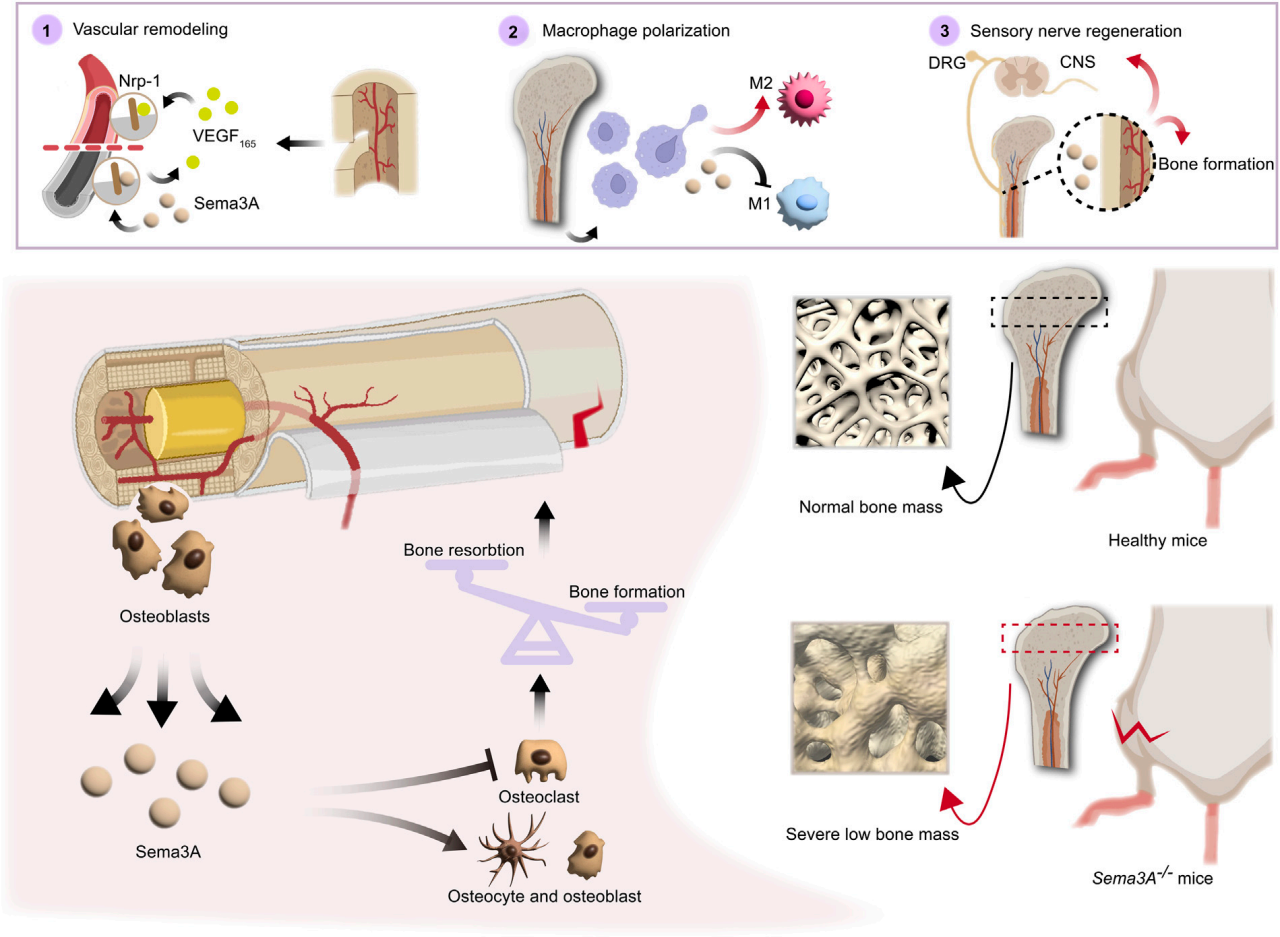
Direct and indirect roles of Sema3A in bone homeostasis. Both directly and indirectly, sema3A, a crucial regulator in the preservation of bone homeostasis, influences bone regeneration. On the one hand, Sema3A acts on bone cells to stimulate bone formation and inhibit bone resorption, thus directly regulating bone repair. On the other hand, via coordinating immunological, vascular, and neurological processes, Sema3A indirectly influences bone formation. In general, Sema3A may inhibit angiogenesis through Nrp-1, while promoting sensory neuron development and modulating macrophage polarization, which create an immune microenvironment conducive to bone repair.

Overall, in synthesis of the data gleaned from studies summarized in Table 1, Sema3A emerges as a multifaceted contributor to bone remodeling by promoting osteoblasts differentiation and inhibiting osteoclast differentiation (Figure 4). The participation of Sema3A in the process of bone formation and resorption is undeniable, despite the conflicting findings of these studies. Additionally, it restricts the migration of Bone marrow-derived monocyte/macrophage precursor cells (BMMs) by reducing RhoA activation (Hayashi et al., 2012). More research is still required to fully understand the connections and mechanisms at work.

**TABLE 1:** Effects of Sema3A on cells involved in bone metabolism.

Study	Methods	External conditions	Results	Mechanisms
Takegahara et al. (2006)	*Plexin-A1* ^*−/−*^mice	*In vivo*	Development of osteopetrosis	Osteoclast numbers**↓**
Hayashi et al. (2012)	*Sema3A* ^*−/−*^ and *Nrp-1* ^*sema-*^ mice	*In vivo*	Bone mass**↓**	Osteoclast formation↑
Hayashi et al. (2012)	1 mg per kg body weight Sema3A	*In vivo*	Osteoblast markers↑ Osteoclast parameters↓	The Wnt pathway Plexin A1-TREM2-DAP12
Fukuda et al. (2013)	2 μg mL^-1^ Sema3A	*In vitro*	Osteoblast differentiation↑	-
Uto et al. (2017)	-	Mechanical repetitive loads (10N, 3 Hz, and 1800 cycles) was exerted twice aweek for 5 weeks	Osteoblasts number↑	-
Azab et al. (2020)	1 μg mL^-1^ Nrp-1	*In vitro*	Osteoclast areas**↓**	The RANKL/RANK pathway
Sun et al. (2021)	2 mL LV-Sema3A	1 μg/ml *E coli* LPS-induced inflammatory environment	Osteogenic differentiation↑	The Wnt/β-catenin pathway
Sen et al. (2021)	10 ng mL^-1^ Sema3A	*In vitro*	hOB differentiation↑	Rac1GTPase the Wnt/β-catenin

Abbreviations: LV-Sema3A, Lentiviral vector-Sema3A; hOB, alveolar bone osteoblasts.

#### 2.1.2 Indirect role of Sema3A in bone homeostasis

Sema3A, expressed in vascular and neural protrusions, emerges as a multifaceted regulator impacting not only bone tissue directly but also bone vascularization and neural invasion, indirectly influencing bone development and remodeling. Upregulation of Sema3A expression is also preferential to the aforementioned processes (Gomez et al., 2005). Given the high vascularity of bone tissues, the vascular system plays a pivotal role in bone growth, healing, and remodeling. Studies on mice lacking Sema3A and Nrp-1 have illuminated the presence of vascular abnormalities in these animals, underscoring the importance of Sema3A in vascular development within bones (Kawasaki et al., 1999).

In a vascular development model, Sema3A-positive cells have been identified as endothelial cells (ECs), and endogenous Sema3A spread on ECs co-localized with focal complexes—a critical location where integrins drive cell migration (Geiger et al., 2001). WOW-1 Fab, a ligand mimicking the ECM, the increase in WOW-1 Fab binding to ECs brought on by either VEGF-A or basic fibroblast growth factor (bFGF) was fully blocked by Sema3A. However, it is interesting to note that *in vivo* overexpression of either Sema3A or Sema3A receptors led to defective vascular remodeling (Serini et al., 2003). Angiogenic growth factors (Byzova et al., 2000) are important determinants of vascular remodeling. Sema3A overexpression is deleterious to tubule formation while it suppresses the expression of endothelial cell markers like CD31 and VEGF-A (Li et al., 2020). The information above implies that Sema3A controls angiomorphogenesis by inhibiting integrin function, this inhibition results from the integrin’s decreased affinity for the ECM ligand, leading to cellular motility on the permissive ECM, which is necessary for angiogenesis. Excess Sema3A has also been linked to endogenous angiogenesis inhibitory molecules. In addition, tumor-associated macrophages (TAMs) are recruited to support tumor progression in avascular regions. However, Sema3A intervenes in this process by initiating the phosphorylation of vascular endothelial growth factor receptor 1 through a related receptor complex involving Nrp1, Plexin-A1, and Plexin-A4. This action prevents macrophages from infiltrating hypoxic tumor regions, thereby inhibiting angiogenesis (Casazza et al., 2013). While it has been established that Plexin-A4 plays a role in inhibiting tumor angiogenesis through Sema3A signaling, it is important to note that there is a need for additional research in the context of vascularization-mediated bone regeneration (Kigel et al., 2011). Interestingly, following total body irradiation (TBI), bone marrow endothelial cells exhibit a significant increase in Sema3A gene expression in response to spinal cord inhibition, Sema3A-Nrp-1 signaling delayed bone marrow vascular regeneration. However, this impact was independent of VEGF (Termini et al., 2021). Thus, Sema3A exhibits a phasic and bipartite role in neovascularization and development.

Moreover, nerves are essential contributors to bone growth and regeneration. Osteogenesis-specific Sema3A-deficient mices exhibit normal bone mass, whereas *Sema3A*_*synapsin*_^*−/−*^ *and Sema3A*_*nestin*_^*−/−*^ mice display reduced bone mass (Fukuda et al., 2013). Sensory nerve fibers, widely distributed in bone tissue, possess the capacity to regulate bone remodeling through the release of neuropeptides like calcitonin gene-related peptide (CGRP), some of which may have implications for blood vessels (Irie et al., 2002). Sema3A has demonstrated the ability to induce sensory neuron death in vitro experiments, and it also triggers apoptosis in sympathetic neurons *in vivo*. These effects are mediated by the binding of Sema3A to Plexin-A3 and Nrp-1 receptors (Wehner et al., 2016). Schwann cells, enveloping sensory neuron axons, express high levels of Sema3A, which acts as an inhibitor of sensory neuron growth by binding to Nrp-1 (Shen et al., 2023). Itriguingly, Zhang et al. (Zhang et al., 2018) discovered that Sema3A had a pro-regenerative effect on corneal sensory nerves following detached dorsal root ganglia (DRG) but did not inhibit NGF-induced nerve growth, suggesting that there may be a means to counteract this developmental inhibitory effect. The intricacies of neural and vascular network invasion in bone remodeling remain a subject of ongoing exploration. In sum, Sema3A plays a multifaceted role in bone development and remodeling through neural-vascular crosstalk (Figure 4).

### 2.2 Bone tissue engineering

Tissue engineering is the application of engineering and biological science principles to the problem of human organ or tissue failure in an effort to repair, maintain, or improve it (Langer and Vacanti, 1993). A spectrum of causes, including trauma, infection, and postoperative tumors, can lead to bone defects, and those that surpass essential dimensions continue to be difficult to treat. Although autologous bone grafting remains the gold standard, its widespread use is marred by limitations, including graft scarcity and postoperative complications after surgery (Dimitriou et al., 2011). The core attributes of bone grafts lie in their capacity for osteoinduction and osteoconduction, vital for stimulating bone growth and providing structural support (Fillingham and Jacobs, 2016). Cell-based and non-cell-based tissue engineering are the two basic categories for bone tissue engineering (Langer and Vacanti, 1993). The former enlist specialized seed cells with the ability to form new bone, such as osteoblasts, BMSCs, and adipose stem cells. In contrast, non-cell-based methodologies harness matrix composites enriched with biomodulatory components to expedite the generation of new tissue upon implantation within the body. Consequently, bone tissue engineering stands as a key method for treating bone defects. Sema3A, recognized as a potent bone protective factor (Hayashi et al., 2012), has garnered substantial attention in bone tissue engineering studies. In this review, we present a compilation of findings from various investigations, shedding light on the utility of Sema3A in bone tissue engineering. Several trials are discussed, and their aggregated results are presented in Table 2.

**TABLE 2:** Application of Sema3A in bone tissue engineering.

Study	Materials	Results	Mechanisms
Liu et al. (2016)	Sema3A/ASCs/PLGA	Bone volume fraction and Bone mineral density↑	Stem cell differentiation, ASCs migration
Li et al. (2020)	Sema3A/HIF1α/HA	ALP, OCN, and OPN↑	iPSC-MSCs differentiation
Song et al. (2022)	Sema3A/Ti	Bone stiffness and toughness↑	Stem cell differentiation
Ma et al. (2022)	Sema3A/Si	ALP and Cell migrated area↑	Angiogenesis, MSCs migration

Abbreviations: A,P: alkaline phosphatase; Ocn: Osteocalcin; Opn, Osteopontin; MSCs, Mesenchymal Stem Cells; ASCs, Adipose Stem cells.

Li et al. (Li et al., 2020) employed induced pluripotent stem cell-derived mesenchymal stem cells (iPSC-MSCs) co-expressing Sema3A and HIF1α within hydroxyapatite (HA) scaffolds, subsequently implanted in a mouse model of cranial bone defects. Notably, elevated mRNA levels of osteogenic gene markers, including bone morphogenetic protein (BMP2), RUNX2, and ALP, were observed, signifying the potential of Sema3A in enhancing proliferation and inducing differentiation of iPSC-MSCs to facilitate cranial bone defect repair. In an osteoporotic rabbit model, Song et al. (Song et al., 2022) reported that the experimental group overexpressing Sema3A had better bone stiffness, toughness, push-out force, and other related evaluation indexes than the control group, implying the positive promotion of Sema3A on early osseointegration of titanium implants, which is also achieved by promoting osteogenic differentiation of stem cells. Ma et al. (Ma et al., 2022) exploited the property of silicon to induce Sema3A expression via sensory nerves to implant silicified collagen scaffolds into a mouse model and assess their efficacy in limiting bone defects. A phenomenon observed in the medium of silicon-stimulated DRG indicates that silicon is capable of promoting the proliferation and migration of MSCs and EPCs, along with angiogenesis. The test group overexpressing Sema3A demonstrated substantial repair of bone defect regions. Lower bone metrics were seen in the control group following the injection of Sema3A-RNAi to reduce Sema3A expression, underscoring Sema3A’s potential role in Si- induced angiogenesis and osteogenesis. Moreover, Liu et al. (Liu et al., 2016) demonstrated that implantation of ASCs-Sema3A into poly (lactic-co-glycolic acid) (PLGA) scaffolds enhances bone repair in a cranial defect model. Notably, Sema3A not only promoted osteogenesis through the Wnt signaling pathway but also upregulated the osteogenic capacity of ASCs *in vivo* by promoting the migration of ASCs to BMSCs. This breakthrough holds promise in addressing the limitations associated with the lower osteogenic differentiation capacity of ASCs.

In conclusion, Sema3A emerges as a multifaceted player in bone tissue engineering, demonstrating its potential to influence multiple facets of bone regeneration. Sema3A’s capacity to promote stem cell migration and differentiation towards bone-forming cells is a pivotal mechanism in bone defect repair. Furthermore, its role in mediating vascularization brought by sensory nerves accelerates the overall healing process. Notably, Sema3A serves as a mediator for osteogenesis facilitated by sensory nerves, further underscoring its relevance in orchestrating bone remodeling and regeneration. The incorporation of Sema3A-modified composites in bone tissue engineering represents a promising and innovative approach that holds the potential for clinical translation in the treatment of bone defects.

### 2.3 Potential roles of Sema3A in bone diseases

#### 2.3.1 Osteoporosis

Osteoporosis, a widespread bone ailment afflicting nearly 10 million people each year in the United States alone (Klibanski et al., 2001). The morbidity and mortality rates of osteoporotic fractures remain high (Johnell and Kanis, 2006). This multifactorial condition arises from various triggers, including aging, hormonal changes, and genetic predisposition (Cummings and Melton, 2002). Crucially, osteoporosis is marked by an imbalance in osteoblast and osteoclast dynamics, along with aberrant apoptosis (Manolagas, 2000). Sema3A, a substance linked to bone homeostasis, might play a role in the control of osteoporosis.

After constructing an adult female mouse ovariectomized (OVX) model of osteoporosis, Yang et al. (Yang et al., 2018) illuminated the potential of Sema3A in addressing osteoporosis by utilizing an osteoporotic mouse model following OVX. Employing a bone-targeted drug delivery system, their study showcased a significant reduction in bone loss in mice with Sema3A overexpression. Additionally, histological analyses revealed an elevated osteoblast count and a concurrent decline in osteoclast numbers, aligning with Sema3A’s demonstrated *in vitro* impact of bolstering osteoblast-mediated bone formation while impeding osteoclast-mediated resorption (Hayashi et al., 2012). Sema3A protein levels significantly increased *in vitro* tests with estradiol (E2)-treated Petri plates, according to Hayashi and coworkers (Hayashi et al., 2019), who reported that Sema3A expression levels decline with age following menopause. This was reconfirmed by *in vivo* experiments in which OVX mice were used to establish an osteoporosis model, and OVX decreased Sema3A expression in bone tissue. However, this inhibitory effect could be counteracted by E2, which was mediated by miRNAs, including miR-4797 and miR-195. It has been proven that Plexin-A2, which functions as an auxiliary receptor to identify Sema3A, has a diversity of gene loci linked to osteoporosis and osteoporotic fractures (Hwang et al., 2006). Interestingly, in a clinical cohort of 860 postmenopausal women, the correlation between Sema3A levels and serum bone formation marker Ocn was affirmed, albeit not with bone mineral density (BMD). Strikingly, Sema3A expression exhibited no significant divergence between fracture and non-fracture groups, challenging prior inferences and underscoring the enigmatic relationship between Sema3A and human bone metabolism.

Osteoporosis remains a significant concern, particularly in scenarios involving excessive glucocorticoid use. A compelling study by Xing and colleagues (Xing et al., 2021) shed light on the impact of Dexamethasone (Dex) on Sema3A expression in BMSCs. Dex exhibited a dose-dependent capacity to diminish Sema3A expression via the PI3K/Akt signaling pathway. This effect was accompanied by reduced nuclear-aggregation of β-catenin and decreased transcriptional activity within BMSCs. Furthermore, Dex impeded osteoblast differentiation, the introduction of exogenous Sema3A effectively counteracted these detrimental outcomes.

In the context of diabetic bone disease, Ma et al. (Ma et al., 2017) made noteworthy observations. They detected a significant reduction in Sema3A expression within bone tissue in a well-established diabetic rat model. This alteration correlated with diminished trabecular number and volume in the femur and lumbar vertebrae, alongside compromised bone formation and mineralization. Of concern, trabecular segregation increased, exacerbating bone fragility and diminishing bone stiffness, which may exert as a potential contributor to diabetic bone disease.

In light of these compelling findings, it becomes apparent that Sema3A may be intricately linked to the risk factors and pathogenesis of osteoporosis. Its multifaceted role in countering skeletal fragility and mitigating bone loss positions Sema3A as a promising candidate for further exploration in the context of therapeutic interventions for osteoporosis.

#### 2.3.2 Osteosarcoma and bone metastases

Sema3A signaling plays a pivotal role in bone tumor progression, and its effects are multifaceted. Notably, Sema3A is implicated in promoting oncogenesis through the mTORC1-mediated Warburg effect (Yamada et al., 2016), and it has also been found to be a target gene significantly associated with the prognosis of osteosarcoma, underscoring its clinical relevance. Furthermore, it has been found that Sema3A gene expression can be upregulated by inhibiting Galectin-1, a pro-tumor vascular factor. This observation hints at a potential link between Sema3A and tumor vascular growth, further expanding its therapeutic implications (Storti et al., 2016). To sum up, Sema3A emerges as a promising therapeutic target in the context of bone tumors and bone metastases. Understanding the intricacies of Sema3A signaling in bone-related cancers is crucial for harnessing its therapeutic potential effectively.

## 3 Roles of Sema3A on cartilage metabolism and diseases

### 3.1 Effects of Sema3A on chondrocytes and cartilage matrix

Healthy human cartilage presents distinct characteristics compared to normal bone tissue. It is characterized by a lower density of chondrocytes, reduced metabolic activity, and a reliance on anaerobic metabolic pathways under low oxygen environments (Tarantino et al., 2021). Cartilage can only be maintained with a normal supply of nutrients under repeated loading, with its primary source of nutrition being synovial fluid as it matures (Ingelmark, 1950). Conventionally, articular cartilage is considered devoid of cellular and humoral immunity, lacks innervation in maturity, and does not rely on nerves for transmitting information. Nevertheless, it is sensitive to mechanical deformation caused by changes in mechanical forces acting on cartilage tissues, which can lead to mechanical signaling and subsequent alterations in metabolic activity (Setton et al., 1999; Kurz et al., 2005). Thus, conditions like trauma, osteoarthritis, and rheumatoid arthritis that compromise cartilage structure can impact normal mechanotransduction and its associated metabolic functions (Shaktivesh et al., 2019).

Emerging evidence underscores the critical role of Sema3A in preserving cartilage health. Adult articular chondrocytes express Sema3A, along with its receptors (Okubo et al., 2011), particularly in the outer perichondrium of the diaphysis and metaphysis. Gomez et al. (Gomez et al., 2005) identified the expression of *SEMA3A* and *NRP1* mRNA in various cartilaginous regions, including the extraepiphyseal cartilage membrane, hypertrophic chondrocytes within primary and secondary ossification centers, and resting chondrocytes. Notably, *Sema3A* ^*−/−*^ mutant mice exhibit abnormalities in both bone and cartilage development (Behar et al., 1996), emphasizing the indispensable regulatory role of the semaphorin family in normal cartilage and bone development.

**FIGURE 5:**
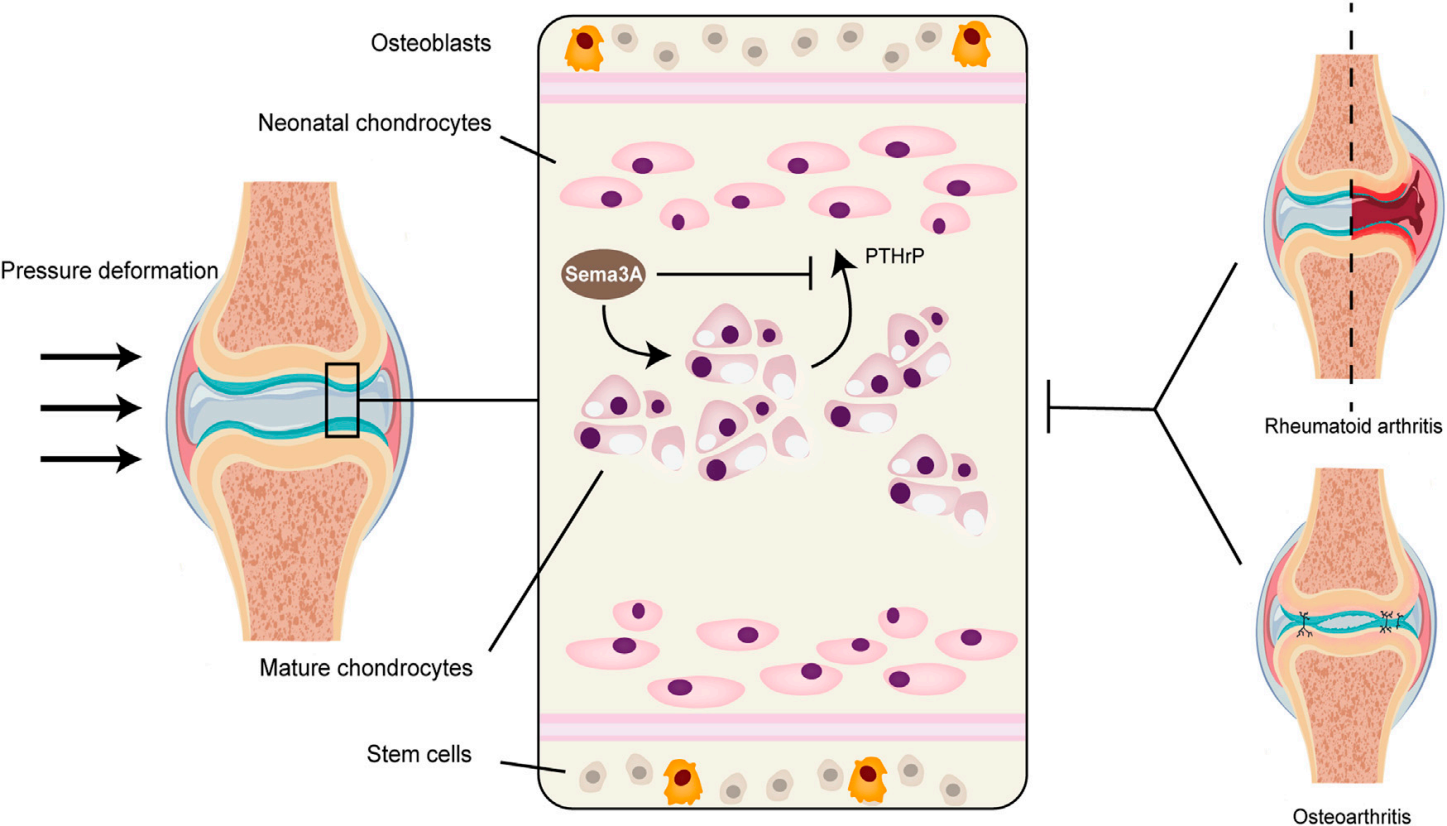
Sema3A signaling in cartilage metabolism and diseases. Because cartilage tissue lacks vascular nerves, its normal metabolism cannot be separated from mechanical signaling. The perichondrium contains the majority of stem cells, and the naive chondrocytes that are close to the perichondrium’s surface gradually multiply and grow into a mature population of homologous chondrocytes. Healthy articular chondrocytes contain Sema3A, which inhibits PTH-R1 and controls chondrocyte proliferation while promoting chondrocyte growth and changing intracellular protein content. Diseases that impact cartilage structure, like rheumatoid arthritis and osteoarthritis, disrupt normal mechanistic signaling, which changes the level of Sema3A expression in the tissue.

Sumi et al. (Sumi et al., 2018) uncovered another facet of Sema3A’s influence, particularly in an inflammatory context with high-amplitude cyclic tension strain (CTS). Here, Sema3A was found to downregulate the activation of traditional inflammatory pathways, such as AKT, ERK, and NF-κB, leading to a reduction in the expression of inflammatory factors in chondrocytes. Moreover, the Sema3A signaling system was implicated in chondrocyte proliferation and chondrogenesis. In culture plates with a 100 mm diameter, the introduction of either low (1 ng/mL) or high (100 ng/mL) concentrations of Sema3A increased the intrachondrocyte protein content and promoted chondrocyte development without altering cell morphology. Exogenous Sema3A also inhibited the intrachondrocyte concentration of PTH-R1, which functions as a PTHrP protein receptor and aids in the regulation of chondrocyte proliferation (Figure 5). This inhibiting impact may hasten the endochondral ossification (Kronenberg, 2003; Kajii et al., 2018). Collectively, these observations suggest that Sema3A plays a pivotal role in mechanotransduction within cartilage, whether under physiological or pathological conditions.

### 3.2 Potential roles of Sema3A in cartilage related diseases

#### 3.2.1 Intervertebral disc degeneration

The intervertebral disc (IVD) undergoes significant changes as it ages, with one notable transformation being the onset of vascular invasion in the outer layer, accompanied by increased expression of inflammatory molecules such as IL-6 (Suzuki et al., 2017). In animal studies, Sema3A expression was found to be higher in the outer annulus fibrosus than in the nucleus pulposus and this expression shows an inverse relationship with the age of rats. Remarkably, Sema3A plays a vital role in maintaining the homeostasis of the intervertebral disc by competitively inhibiting VEGF165, which in turn inhibits angiogenesis. Additionally, Sema3A expression is downregulated by IL-6 via the JAK/STAT3 signaling pathway (Mima et al., 2019), resulting in decreased Sema3A cell immunopositivity in degenerated IVDs. Overall, these findings underscore the critical role of Sema3A in preserving disc homeostasis and delaying degenerative processes.

#### 3.2.2 Rheumatoid arthritis

Rheumatoid arthritis (RA), a chronic autoimmune disease, is characterized by persistent inflammation of the synovial membrane in joints, leading to the invasion of articular cartilage, subchondral bone, and periarticular tissues. Recent investigations have unveiled Sema3A’s involvement in RA pathology and immunological regulation.

Animal studies revealed that Sema3A alleviates symptoms in mice with collagen-induced arthritis (CIA) (Catalano, 2010). In comparison to healthy groups, RA patients exhibited considerably greater serum Sema3A expression. Inflammatory markers (e.g., RF, ESR, IgM), RA autoantibodies (e.g., anti-CCP, APF, GPI), lumbar spine BMD, and disease activity were all positively linked with serum Sema3A levels (Gao et al., 2018). However, Catalano et al. (Catalano, 2010) noted a temporary deficiency in Sema3A expression in CD4^+^ T cells from RA patients through an unidentified mechanism. Inflammation plays a pivotal role in RA progression, and increasing attention is being drawn to the inflammation-hypoxia-angiogenesis axis. Hypoxia-inducible molecules, including VEGF, contribute to the inflammatory infiltrate and the generation of other mediators (Konisti et al., 2012). Gerber et al. (Gerber et al., 1999) highlighted that capillary invasion regulated by VEGF triggers cartilage remodeling. When VEGF is deactivated in mice, chondrocyte resorption is halted. Sema3A is expressed on macrophages and promotes M1-type macrophages (pro-inflammatory) induced by the LPS/IFN-γ to IL-4-induced M2-type macrophages (anti-inflammatory) (Ji et al., 2009) (Figure 4). Furthermore, it has been observed that the expression of Sema3A receptors, including Nrp-1, Nrp-2, Plexin-A1, Plexin-A2, and Plexin-A3, is substantially upregulated during M-CSF-mediated macrophage polarization, leading to the selective activation of the M2 phenotype (Ji et al., 2009). These findings highlight the intricate involvement of Sema3A and its receptors in the regulation of macrophage phenotypes, particularly favoring the anti-inflammatory M2 subtype. Additionally, Sema3A inhibited VEGF-induced endothelial cell proliferation and migration (Teng et al., 2017). Sema3A may play a moderating role in the onset of cartilage degradation and inflammation in RA, although its mediation of the effect of VEGF on chondrocytes remains elusive.

Synovial hyperplasia is another hallmark of RA, where fibroblast-like synoviocytes (FLS) accumulate in the synovial mechanism and produce cartilage-eroding enzymes like MMPs (Bottini and Firestein, 2013). Sema3A has been shown to enhance FLS migration, invasion, and upregulation of *MMP8* mRNA. Thus, the deterioration of joints in RA appears to be mediated by Sema3A. Of note, early RA patients exhibit lower Sema3A expression in synovial tissues (Tang et al., 2018). While current research indicates that Sema3A mitigates the inflammatory response in RA, further research are warranted to elucidate its impact on cartilage.

#### 3.2.3 Osteoarthritis

Osteoarthritis (OA) is a chronic degenerative condition characterized by the gradual loss of articular cartilage, accompanied by bone regeneration in the articular rim and subchondral bone, with cartilage often being the initial site of deterioration. Secondary OA, frequently arising from cartilage damage, accelerates OA development by disrupting the anabolic balance between chondrocytes and the extracellular matrix (Altman et al., 1986). Recent studies have confirmed that Sema3A is involved in the degenerative loss of OA cartilage.

Okubo et al. (Okubo et al., 2011) observed higher concentrations of Sema3A in cartilage from OA patients, and it was hypothesized that its role in chondrogenic cloning in OA lesions. This effect appears to be related to VEGF, known to promote chondrocyte migration without impacting proliferation. Interestingly, chondrocyte migration was only inhibited when Sema3A and VEGF165 were co-treated with chondrocyte culture dishes, and the Sema3A-mediated inhibition of VEGF165 was blocked by the addition of SM216289, a Sema3A inhibitor, suggesting that Sema3A plays a role in chondrocyte clones in OA cartilage by inhibiting cell migration. Furthermore, Stoeckl et al. (Stoeckl et al., 2022) identified that Sema3A could exert a catabolic effect on OA chondrocytes by upregulating the expression of MmP13, while downregulating the activation of AKT, which can maintain chondrocyte survival. Inhibition Nrp-1 decreased the expression of Mmp13, enhanced adhesive capacity, and increased a senescence marker SA-β-galactosidase activity in OA chondrocytes. However, it had no influence on apoptosis or migration. In a different vitro experiment, exogenous Sema3A treatment significantly decreased the number of chondrocytes, indicating both a pro-regulatory effect of Sema3A and its high dependence on Nrp-1. However, overexpression of Nrp-1 signaling had no discernible regulatory effect on cell proliferation or apoptosis (Sun et al., 2018).

Beyond its impact on local chondrocytes and cellular matrix of the joint, the release of Sema3A from cartilage through mechanotransduction signals may also affect the structural elements of the periarticular vascular and neural tissues. Gomez et al. (Gomez et al., 2005) found the presence of two neuromarkers, neurofilament heavy chain (NH) and tyrosine hydroxylase (TH), in epiphyseal chondrocyte membranes, corresponding with the spatial and temporal distribution of Sema3A and its co-receptors. Despite the absence of vascular innervation of articular cartilage, Sema3A seems to be temporally and spatially associated with the invasion of bone by vascular and nerve fibers. Researches has validated the connection of SP and CGRP with OA-related pain (Benschop et al., 2014; Warner et al., 2017). Interestingly, Sema3A is a selective repellent of SP and αCGRP-positive sensory nerve fibers (Stoeckl et al., 2022). These evidences suggest that Sema3A may affect pain in OA patients through neural interactions.

## 4 Conclusion and future directions

Existing researches have confirmed that the widespread expression of Sema3A and its receptors in bone cells and chondrocytes, implicating their involvement in bone and cartilage metabolism through various signaling pathways. Moreover, Sema3A’s function in cartilage metabolism and the utility of using it in bone tissue engineering are specifically summarized for the first time in this paper.

However, it is important to acknowledge that while Sema3A has shown osteoprotective effects, the specific mechanisms of Sema3A on the human skeleton remain insufficiently explored. The majority of the current experiments are still focused on animal models, predominantly in rodents. It is crucial to recognize that the effects of Sema3A on rodent bones may differ from those in humans. Therefore, to fully understand the impact of Sema3A in the context of the human body, considering the immune system and nervous system, and to assess its potential as a therapeutic agent for bone diseases, more clinical data on bone diseases are needed. In addition, the pro-apoptotic effect of Sema3A on cells such as BMCs further limits its value for clinical applications, necessitating further research to address this challenge.

Certainly, recent theories propose indirect regulation through the vasculature, independent of VEGF, whereas it was previously believed that the inhibitory effect of Sema3A on the vasculature was achieved through VEGF. Nrp-1 acted as a co-receptor for both VEGF and Sema3A, but it has now been suggested that this potency may be independent of VEGF and primarily mediated by Nrp-1. Furthermore, despite its conventional role as a neuron growth inhibitor, Sema3A may have mutagenic effects on sensory nerve sprouting and facilitate the recovery of peripheral sensory nerves (Zhang et al., 2018).

Sema3A regulate chondrocyte migration, proliferation, adhesion, and other works, which in turn affect cartilage mechanical signaling and pathological processes. There have not been numerous studies on Sema3A’s function in cartilage metabolism, and the results of those studies have not all been agreed upon, as evident in the conflicting effects of Sema3A on chondrocyte survival in different conditions.
